# Operational Momentum in Multiplication and Division?

**DOI:** 10.1371/journal.pone.0104777

**Published:** 2014-08-14

**Authors:** Curren Katz, André Knops

**Affiliations:** Faculty of Life Sciences, Humboldt Universität zu Berlin, Berlin, Germany; Center for BrainHealth, University of Texas at Dallas, United States of America

## Abstract

Biases are commonly seen in numerical cognition. The operational momentum (OM) effect shows that responses to addition and subtraction problems are biased in the whole-number direction of the operation. It is not known if this bias exists for other arithmetic operations. To determine whether OM exists in scalar operations, we measured response bias in adults performing symbolic (Arabic digits) and non-symbolic (dots) multiplication and division problems. After seeing two operands, with either a multiplication (×) or division (**÷**) sign, participants chose among five response choices. Both non-random performance profiles and the significant contribution of both operands in a multiple regression analysis predicting the chosen values, suggest that adults were able to use numerical information to approximate the outcomes in both notations, though they were more accurate on symbolic problems. Performance on non-symbolic problems was influenced by the size of the correct choice relative to alternatives. Reminiscent of the bias in addition and subtraction, we found a significant response bias for non-symbolic problems. Non-symbolic multiplication problems were overestimated and division problems were underestimated. These results indicate that operational momentum is present in non-symbolic multiplication and division. Given the influence of the size of the correct choice relative to alternatives, an interaction between heuristic bias and approximate calculation is possible.

## Introduction

Humans, as well as other animals, have an innate approximate number sense that they use to interact with the environment [1]–[3]. Disease, injury [4], and environmental variables (*e.g.* ineffective education) can impact this system at a high cost to individuals and society. Even in healthy populations, lower numeracy predicts poor decision making and susceptibility to bias [5]. Given the importance of numerical abilities, research has focused on understanding the underlying cognitive processes.

According to the *triple-code model*, numbers can be represented in three codes [6]. In the Arabic code, associated with bilateral occipito-temporal regions, numbers are represented as Arabic numerals and can be used to perform symbolic arithmetic. In the verbal code, associated with left perisylvian language areas, numbers are represented as words and memorized arithmetic facts. In the magnitude code, associated with bilateral parietal areas, numbers are represented as abstract magnitudes and perhaps points on a spatially oriented mental number line (MNL). Consistent with the idea of a mental number line, parietal neural populations tuned to small quantities exhibit a topographic organization [7]. This innate approximate number system (ANS) supports quantity knowledge (*e.g.* 3 is smaller than 7), as well as estimation and calculation on non-symbolic quantities [8].

Perhaps due to the spatial features of quantity representation, spatial and directional biases are frequently seen in numerical tasks. The *Spatial-Numerical Association of Response Codes* (SNARC) effect shows that smaller numbers are left-side associated and larger numbers are right-side associated [9], [10]. Further evidence for the spatial nature of number representation comes from magnitude-dependent covert shifts of attention during number viewing [11], [12]. Directional bias is seen in addition and subtraction, when participants overestimate for addition and underestimate for subtraction [13]. This operational momentum (OM) effect occurs in adults performing non-symbolic and, to a lesser extent, approximate symbolic arithmetic [14]. Infants exhibit OM as well, demonstrated by looking longer at arithmetic animations violating the momentum of the operation [15]. Interestingly, school-age children may overestimate non-symbolic subtraction, although this could be due to individual differences in attention [16]. OM occurs in exact symbolic arithmetic, as long as an approximate response method is used [17], [18].

Although OM research has focused on whole numbers, adults and children answering symbolic arithmetic questions also show a tendency to believe addition/multiplication always makes more than the initial quantity and subtraction/division always makes less, even though this is not necessarily true with operations including non-whole rational numbers (*e.g*. 8×.5 = 4) or zero (*e.g.* 8×0 = 0) [19], [20]. The origin of this whole-number bias is still a matter of debate [21]. In all four arithmetic operations, the ‘addition/multiplication makes bigger, subtraction/division makes smaller’ intuition [22] could lead to the correct choice, over/under-estimation in the direction of the operation (an OM effect), or even over/under-estimation counter to the direction of the operation (a reverse OM effect), as long as the estimation was larger than the initial quantity. OM research demonstrating systematic over and under estimation on approximate symbolic addition and subtraction problems shows that, at least for these operations, whole numbers themselves are subject to directional biases. It is not yet known if whole-number multiplication and division are subject to directional biases.

Different explanations for OM have been proposed based on response bias in addition and subtraction. Addition and subtraction have been described as spatial movements on a mental number line [23], and the OM effect attributed to movements or shifts of attention too far along this line [14], [24]. Alternatively, if the mental number line is logarithmically compressed [8], [25], OM may result from flawed decompression [13], though not all OM research has supported this [16], [17]. A simple rule of accepting more than the original operand for addition and less for subtraction may also explain the observed bias [15], [19]–[21]. Whether these explanations for OM can be reconciled remains unclear. Since the term operational momentum could imply a spatial origin of the observed bias, it is important to separate proposed cognitive underpinnings (*e.g.* spatial, attentional shifts, etc.) from the observed bias. When we use the term OM, we refer to the observed empirical response pattern without any assumptions about the underlying mechanism [26], [27].

Although a fair amount of research has focused on OM in addition and subtraction, scalar operations such as multiplication and division have never been tested. In this article, “scalar operations” refer to problems where a quantity element is modified by a scalar element [45]. Studies of other operational biases have only used symbolic formats [19], [20]. This may be due to the small number of studies addressing non-symbolic scalar operations, most of which have focused on children prior to instruction [28], [29]. Children in kindergarten and 1^st^ grade can double and halve discrete (dot arrays) and continuous (lines) stimuli [28]. Children in this age group are also able to quadruple and even multiply by a fraction (*e.g.* 2.5) [29]. The limited existing research supports non-symbolic multiplication and division ability and therefore the possibility of studying OM in these operations. Demonstration of OM in scalar operations would add to our understanding of OM in particular and numerical decision making in general.

In this context, we designed a study to test whether OM exists in whole-number multiplication and division by presenting symbolic and non-symbolic problems and measuring response bias. Our first goal was to see whether OM exists in whole-number multiplication and division. Finding OM in multiplication and division could suggest that the ANS influences scalar operations. Our second goal was to see whether participants could use the ANS to solve non-symbolic multiplication and division problems using larger quantities (operands and results) than previous studies. We found that participants based their responses on a combination of both operands, implying reliance on numerical information rather than mere guessing or plausibility checks. Most importantly, they demonstrated OM in non-symbolic problems.

## Materials and Methods

### Ethics Statement

The study was approved by the Humboldt University Department of Psychology Ethics committee (Nr.: 2010–12) on October, 8, 2010. Written informed consent was obtained. Participants were reimbursed 8€/hour for participation in the study.

### Participants

Sixteen native German-speaking right-handed participants (12 female; 20–65 years old, mean = 33.88, SD = 13.12) were recruited in Berlin, Germany, using a Humboldt University department database. Participants who reported a history of psychiatric illness were excluded.

### Stimuli

**Figure 1:**
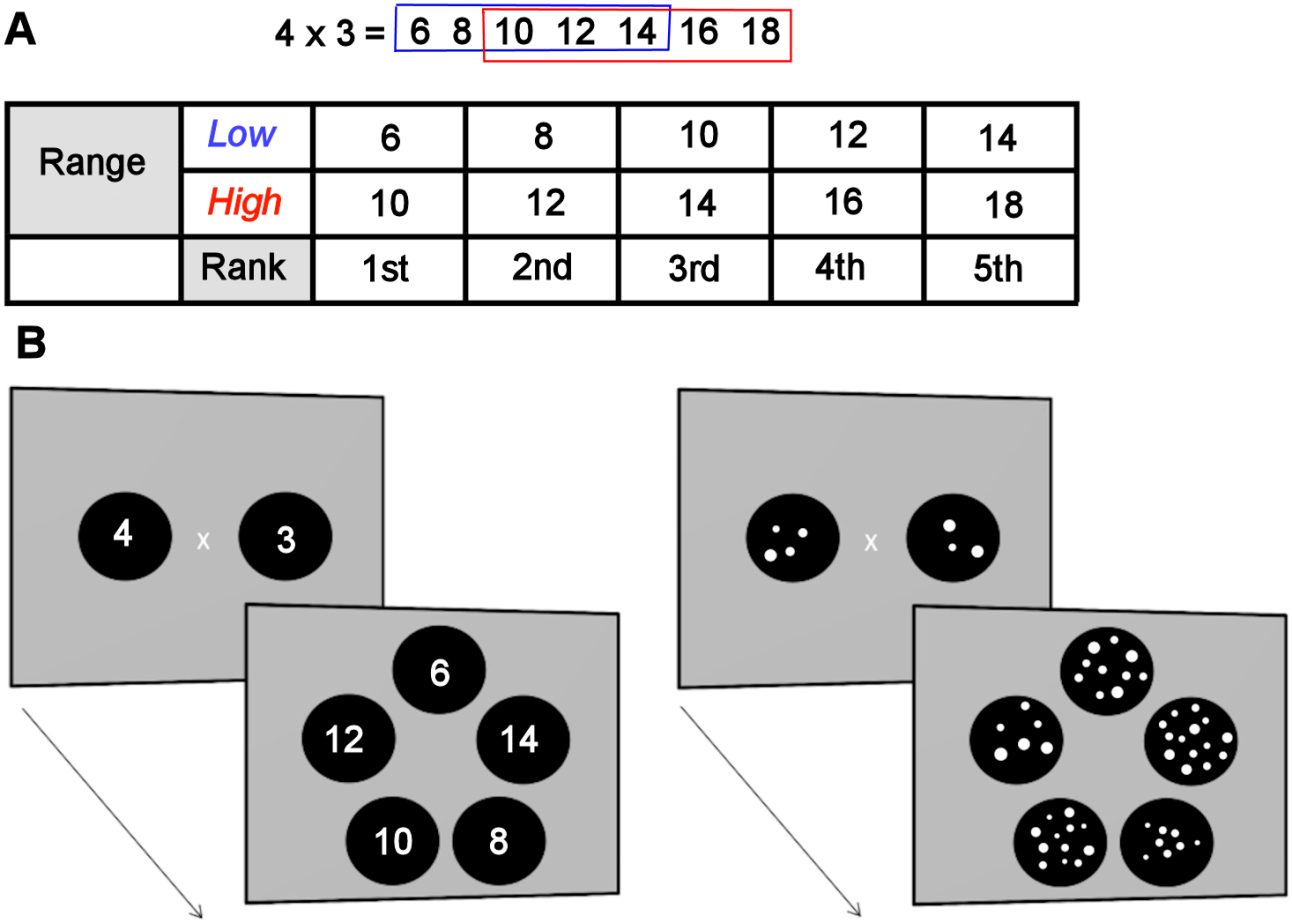
Task design. The correct result (C) and 6 incorrect results were created in a geometric series (symbolic: C x *1.5 ^i/3^* & non-symbolic: *C x 2 ^i/3^*; *i* from −3 to 3). To avoid the correct result corresponding to the ‘middle one’ among presented answer choices, only 5 of the 7 results were presented in a given trial. (A) A low range (blue) with the 4^th^ choice correct and a high range (red) with the 2^nd^ choice correct were created. (B) Illustration of the procedure using 4×3 low (4^th^ choice correct), in symbolic (left) and non-symbolic (right) format. The problem was presented horizontally for 3s, followed by the answer choices for a maximum of 4s. Responses were made with a mouse.

Twenty-four multiplication and 24 division problems were created (table 1). To control for correct value size, the same response choices, including dot arrays for non-symbolic problems, were used for multiplication and division. The task design was based on a previously reported adult OM assessment method [14], [16]. The correct result (*C*) and 6 incorrect results were created in a geometric series (symbolic: C x *1.5 ^i/3^* & non-symbolic: *C x 2 ^i/3^*; *i* from −3 to 3). Previous research has shown that subjects tend to avoid extreme results in symbolic calculation [14]. To increase the likelihood of finding an OM effect in symbolic problems, 1.5 rather than 2 was used. To control for parity, symbolic response alternatives were rounded to the closest value with the same parity as the correct result. To avoid the strategy of choosing the middle value, only 5 of the 7 possible results were presented. In 50% of trials the low range was presented and the 4^th^ result was correct. In the other 50% of trials the high range was presented and the 2^nd^ result was correct (Fig. 1).

**Table 1:** Multiplication and division problems and response choice values.

×	÷	Response Choice Values													
		*Symbolic*							*Non-symbolic*						
		*Correct* x 1.*5^i^* ^/3^							*Correct x* 2*^i^* ^/3^						
*i*		−3	−2	−1	0	1	2	3	−3	−2	−1	0	1	2	3
4×3	48/4	6	8	10	**12**	14	16	18	6	8	10	**12**	16	20	24
6×3	36/2	12	14	16	**18**	22	24	26	9	12	14	**18**	22	28	36
7×3	63/3	13	17	19	**21**	23	27	31	11	13	17	**21**	27	33	41
8×3	78/2	16	18	22	**24**	28	32	36	12	16	20	**24**	30	38	48
9×3	54/2	19	21	23	**27**	31	37	41	13	17	21	**27**	35	43	53
6×4	96/4	16	18	22	**24**	28	32	36	12	16	20	**24**	30	38	48
7×4	112/4	18	22	24	**28**	32	36	42	14	18	22	**28**	36	44	56
8×4	128/4	18	24	28	**32**	36	42	48	16	20	26	**32**	40	50	64
9×4	144/4	24	28	32	**36**	42	48	54	18	22	28	**36**	46	58	72
7×6	126/3	28	32	36	**42**	48	56	62	22	26	34	**42**	52	66	84
8×6	192/4	32	36	42	**48**	54	62	72	24	30	38	**48**	60	76	96
9×6	162/3	36	42	48	**54**	62	72	82	26	34	42	**54**	68	86	108
12×3	108/3	24	28	32	**36**	42	48	54	18	22	28	**36**	46	58	72
14×3	168/4	28	32	36	**42**	48	56	62	22	26	34	**42**	52	66	84
16×3	144/3	32	36	42	**48**	54	62	72	24	30	38	**48**	60	76	96
17×3	153/3	31	39	47	**51**	59	67	79	25	33	41	**51**	65	81	101
19×3	171/3	37	43	49	**57**	63	73	87	29	35	45	**57**	71	91	113
12×4	96/2	32	36	42	**48**	54	62	72	24	30	38	**48**	60	76	96
13×4	156/3	34	38	46	**52**	58	68	78	26	32	42	**52**	66	82	104
16×4	128/2	42	48	56	**64**	74	84	96	32	40	50	**64**	80	102	128
17×4	136/2	46	52	54	**68**	78	92	102	34	42	54	**68**	86	108	136
19×4	152/2	52	58	68	**76**	86	98	114	38	48	60	**76**	96	120	152
13×6	156/2	52	58	68	**78**	92	102	118	38	50	62	**78**	98	124	156
14×6	168/2	56	64	74	**84**	96	112	126	42	52	66	**84**	106	134	168

Response choice values were rounded to match parity of the correct choice value.

Non-symbolic stimuli were created using MATLAB (The MathWorks, Inc., 2012) and the Psychophysics Toolbox extension [30], [31], using the method described by Gebuis and Reynvoet [32]. Previous research has varied intensive (*e.g.* dot size) and extensive (*e.g.* envelope size, area, density) parameters separately. In this case, although participants cannot rely on one feature for all trials they could, for example, use area in half the trials to accurately predict quantity and dot size in the other trials, choosing the best strategy for each trial. We overcame this by first generating 2 dot arrays for each of the five response choice values (comp_dots_version180112.m, http://titiagebuis.eu). We then selected an optimal combination of 5 dot arrays by testing the correlation of visual parameters and quantity for all possible combinations. We chose combinations with individual correlations less than.4 to create groups of uncorrelated dot arrays. The mean correlations between quantity and extensive and intensive visual parameters were 0.05 and 0.02, respectively (area subtended: mean r = 0.05, SD = 0.21; mean dot size: mean r = 0.02, SD = 0.19).

### Procedure

The task was created and presented using OpenSesame [33]. A total of 384 trials were presented in 16 blocks with 24 calculation trials (12 high range & 12 low range) per block. Breaks were given between blocks. Operands were presented simultaneously to reduce working memory confounds. The problem was shown horizontally for 3s with either a multiplication (×) or division (÷) sign between the operands, followed by a screen with the 5 response choices arranged in a circle (Fig. 1). Responses were made using a mouse. The task advanced after a response was made or after a maximum of 4 seconds. The participants were told to answer quickly, even if they were not certain of the exact answer, and not to count the dots in non-symbolic problems.

### Analysis

Data were visualized and analyzed using SPSS 20. To confirm that response choices were not random and check for a significant response bias, repeated measures ANOVA was used. Since interpretation of main effects in the presence of a significant interaction is not recommended [34], simple effects analysis was used when a significant interaction was present. The Bonferroni method was used to correct for multiple comparisons. When Mauchley’s test of sphericity indicated that the assumption of sphericity had been violated, the Greenhouse-Geisser correction was used. Consistent with the notion of a logarithmically compressed mental magnitude representation and previous research [14], correct and response values were log-transformed prior to ANOVA [35].

## Results

### Nonrandom distribution of responses

To investigate the effects of notation, operation, rank (1–5) and range (4^th^ or 2^nd^ choice correct) on response percentage, a series of repeated measures ANOVAs were used. The correct choice can only be inferred using both range and rank variables. Thus, since response percentage results are only meaningful when both variables are considered, *interactions* including only one of these variables were not included. There was a significant interaction between notation, operation, rank and range (*F*(4, 60) = 7.802, *p*<.001, partial η^2^ = .342), qualifying other main and lower-order interaction effects (notation × range × rank: *F*(4, 60) = 259.672, *p*<.001, partial η^2^ = .945; operation × range × rank: *F*(4, 60) = 5.488, *p* = .001, partial η^2^ = .268; range × rank: *F*(4, 60) = 251.132, *p*<.001, partial η^2^ = .944; notation: *F*(1, 15) = 6.808, *p* = .020, partial η^2^ = .312; operation: *F*(1, 15) = 1.676, *p* = .215, partial η^2^ = .100; range: *F*(1, 15) = 1.436, *p* = .249, partial η^2^ = .087; rank: *F*(4, 60) = 84.525, *p*<.001, partial η^2^ = .849). Following up on the significant four-way interaction, we submitted response percentages to 2×5×2 repeated measures ANOVAs, separately for symbolic and non-symbolic notations, with the factors operation (multiplication vs. division), rank (1–5), and range (4^th^ or 2^nd^ choice correct). In symbolic problems, there was a significant interaction between operation, range and rank (*F*(4, 60) = 17.054, *p*<.001, partial η^2^ = .532), qualifying other main and lower-order interaction effects (range × rank: *F*(4, 60) = 333.555, *p*<.001, partial η^2^ = .957; operation: *F*(1, 15) = 4.776, *p* = .045, partial η^2^ = .242; range: *F*(1, 15) = 2.020, *p* = .176, partial η^2^ = .119; rank: *F*(4, 60) = 218.421, *p*<.001, partial η^2^ = .936). In non-symbolic problems, there was no significant interaction between operation, range, and rank (*F*(4, 60) = .978, *p* = .427, partial η^2^ = .061). The interaction between range and rank for all non-symbolic problems was significant (*F*(4, 60) = 31.626, *p*<.001, partial η^2^ = .678), qualifying the main effects (operation: *F*(1, 15) = 4.655, *p*<.048, partial η^2^ = .237; range: *F*(1, 15) = .075, *p*<.787, partial η^2^ = .005; rank: *F*(4, 60) = .334, *p* = .854, partial η^2^ = .022). Therefore, we analyzed the impact of rank and range using 2-way repeated measures ANOVAs, separately for each combination of notation and operation.

### Influence of rank and range on response percentage

**Figure 2:**
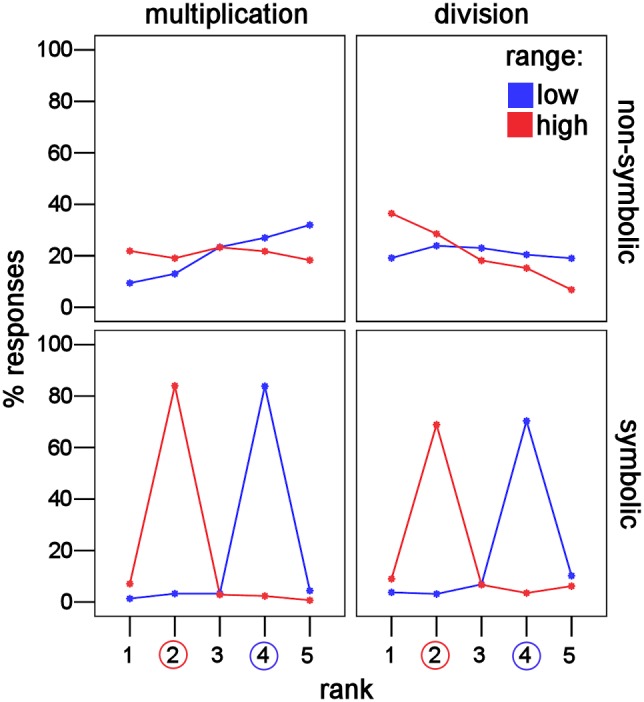
Non-random distribution of responses. Symbolic response percentages were non-random and peaked at the correct result. Rank (1–5) had a significant effect on response percentage for both low (blue, 4^nd^ correct) and high (red, 2^nd^ correct) ranges. Non-symbolic responses were non-random, depending on the response range presented. Rank had a significant effect on response percentage for multiplication when the low (blue) range was presented and for division when high (red) range was presented. This indicates that subjects were not guessing, but rather using a calculation based strategy.

We first checked whether responses were non-randomly distributed, to confirm that participants were not guessing. Since five possible answer choices were used, with the rank of the correct choice depending on the range presented, random responding would be a flat line for both low and high ranges, with 20% of responses in each of the five choices. Based on visual inspection, responses appeared non-random in all conditions (Fig. 2). In symbolic problems, participants chose the correct answer 87% of the time for multiplication (4^th^ choice correct: 1^st^ = 1%, 2^nd^ = 3%, 3^rd^ = 3%, 4^th^ = 87%, 5^th^ = 5%; 2^nd^ choice correct: 1^st^ = 7%, 2^nd^ = 87%, 3^rd^ = 3%, 4^th^ = 2%, 5^th^ = 1%) and around 74% for division (4^th^ choice correct: 1^st^ = 4%, 2^nd^ = 3%, 3^rd^ = 7%, 4^th^ = 75%, 5^th^ = 11%; 2^nd^ choice correct: 1^st^ = 10%, 2^nd^ = 73%, 3^rd^ = 7%, 4^th^ = 4%, 5^th^ = 7%). In non-symbolic multiplication, participants chose close to the correct answer, with a trend towards overestimation, when the 4^th^ choice was correct (1^st^ = 9%, 2^nd^ = 12%, 3^rd^ = 22%, 4^th^ = 26%, 5^th^ = 31%), but chose randomly when the 2^nd^ choice was correct (1^st^ = 21%, 2^nd^ = 18%, 3^rd^ = 22%, 4^th^ = 21%, 5^th^ = 18%). In non-symbolic division, the opposite trend was seen. Participants chose randomly when the 4^th^ choice was correct (1^st^ = 18%, 2^nd^ = 23%, 3^rd^ = 22%, 4^th^ = 19%, 5^th^ = 18%), but chose close to the correct answer, with a tendency towards underestimation, when the 2^nd^ choice was correct (1^st^ = 35%, 2^nd^ = 27%, 3^rd^ = 17%, 4^th^ = 14%, 5^th^ = 6%).

This was confirmed using two-way repeated measures ANOVAs, separately for each condition, with response percentage as the dependent variable and rank of the response choice (1–5) and range (high: 2^nd^ or low: 4^th^ choice correct) as factors. When a significant interaction was present, simple effects analysis was performed to see whether rank had an effect on response percentage, separately for low and high ranges.

#### Symbolic multiplication

Responses were non-random regardless of range of response choices presented. The assumption of sphericity was violated according to Mauchley’s Test of sphericity, χ^2^(9) = 65.378, *p*<.001; therefore degrees of freedom were corrected using Greenhouse-Geisser estimates of sphericity. The interaction between rank and range on response percentage was significant (*F*(4, 60) = 421.783, *p*<.001, partial η^2^ = .966, ε = .312), qualifying significant main effects (range: *F*(1, 15) = 2.246, *p* = .155, partial η^2^ = .130; rank: *F*(4, 60) = 332.318, *p*<.001, partial η^2^ = .957). Therefore, a simple effects analysis was performed. There was a statistically significant difference in response percentage between the five response choices for both 2^nd^ (partial η^2^ = .963) and 4^th^ (partial η^2^ = .968) choice correct trials (table 2).

**Table 2:** Effect of response choice rank on response percentage for low and high range.

					*Sphericity*	
Range	df	F	*P*	Partial 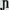 ^2^	χ^2^	ε
*Symbolic multiplication*						
Low, 4^th^ correct*	4,60	452.392	<.001	.968	69.344*	.333
High, 2^nd^ correct*	4,60	391.147	<.001	.963	92.150*	.292
*Non-symbolic multiplication*						
Low, 4^th^ correct*	4,60	11.460	<.001	.433	27.291*	.453
High, 2^nd^ correct	4,60	0.777	.460	.049	29.160*	.463
*Symbolic division*						
Low, 4^th^ correct*	4,60	142.784	<.001	.905	75.556*	.305
High, 2^nd^ correct*	4,60	153.190	<.001	.911	69.293*	.313
*Non-symbolic division*						
Low, 4^th^ correct	4,60	0.839	.447	.053	28.050*	.527
High, 2^nd^ correct*	4,60	16.979	<.001	.531	35.074*	.475

Bonferroni corrected for multiple comparisons and Greenhouse-Geisser corrected for violations of sphericity as measured by Mauchley’s Test of Sphericity.

*p<.001.

#### Non-symbolic multiplication

Responses were not random but, unlike symbolic calculations, this depended on the range of response choices presented. Responses where non-random when the 4^th^ choice was correct but random when the 2^nd^ choice was correct. The interaction between rank and range on response percentage was significant (*F*(4,60) = 13.667, *p*<.001, partial η^2^ = .477), qualifying the significant main effects (range: *F*(1, 15) = .024, *p* = .879, partial η^2^ = .002; rank: *F*(4, 60) = 4.648, *p* = .002, partial η^2^ = .237). Therefore, a simple effects analysis was performed. There was a statistically significant difference in response percentage between the five response choices when the 4^th^ (partial η^2^ = .433) rather than the 2^nd^ choice was correct (partial η^2^ = .049) (table 2).

#### Symbolic division

Like symbolic multiplication, responses were not random regardless of the range of response choices presented. The assumption of sphericity was violated according to Mauchley’s Test of sphericity, χ^2^(9) = 66.359, *p*<.001; therefore degrees of freedom were corrected using Greenhouse-Geisser estimates of sphericity. The interaction between rank and range on response percentage was significant (*f*(4,60) = 188.257, *p*<.001, partial η^2^ = .926, ε = .317), qualifying significant main effects (range: *F*(1, 15) = .135, *p* = .718, partial η^2^ = .009; rank: *F*(4, 60) = 101.163, *p*<.001, partial η^2^ = .871). Therefore, a simple effects analysis was performed. There was a statistically significant difference in response percentage between the five response choices for both 2^nd^ (partial η^2^ = .911) and 4^th^ (partial η^2^ = .905) choice correct trials (table 2).

#### Non-symbolic division

Responses were not random but, similar to non-symbolic multiplication, this depended on the response range presented. In contrast to non-symbolic multiplication, responses were non-random when the 2^nd^ rather than the 4^th^ choice was correct. The interaction between rank and range on response percentage was significant (*F*(4,60) = 18.765, *p*<.001, partial η^2^ = .556), qualifying significant main effects (range: *F*(1, 15) = .135, *p* = .718, partial η^2^ = .009; rank: *F*(4, 60) = 6.827, *p*<.001, partial η^2^ = .313). Therefore, a simple effects analysis was performed. There was a statistically significant difference in response percentage between the five response choices for 2^nd^ choice correct trials (η^2^ = .531), but not 4^th^ choice correct trials (partial η^2^ = .053) (table 2).

### Linear increase of response value with correct value

**Figure 3:**
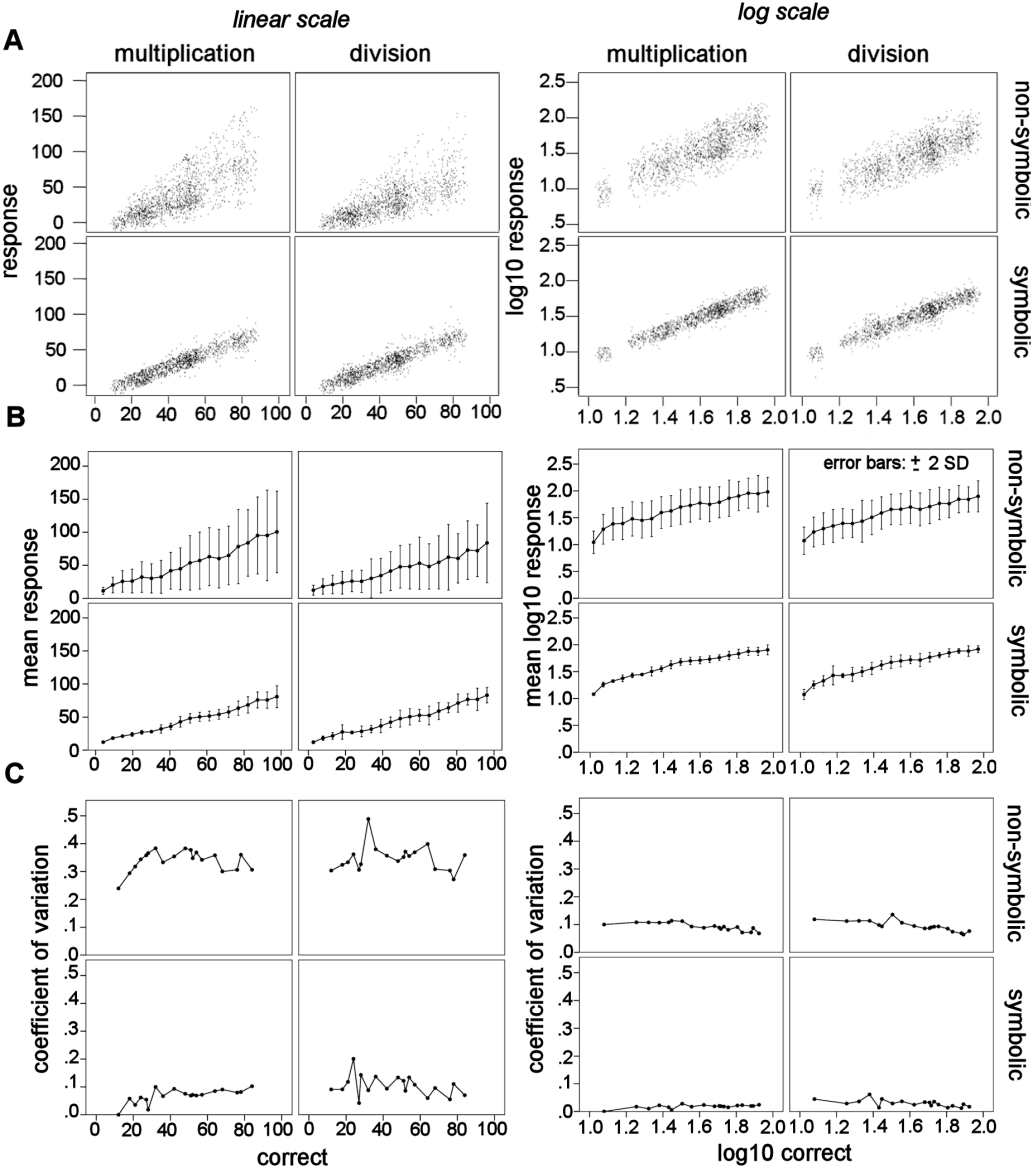
Response value as a function of correct value on linear and log-scale data. (A) Non-aggregated response value as a function of correct value, on the linear (left) and log (right) scale. Number of cases is shown by increased density (*i.e.* darker color). (B) Linear response value and SD increased as a function of correct value, consistent with Weber’s law. Log-transformed response value, but not SD, increased as a function of log-transformed correct value (*i.e.* linear on the log scale). (C) Dispersion of response choices, measured by the coefficient of variation, was constant across correct values on the log scale, but not on the linear scale. Dispersion was constant when log-transformed values were used.

Before analyzing response bias, we wanted to determine whether the logarithm of response and correct values should be used as in past research [14], [16], and in-line with statistical recommendations [35]. Weber’s law predicts that the variability of response values will increase with numerical magnitude. On the linear scale, mean response value and variability increased as a function of the correct value, whereas on the log scale, variability was constant (Fig. 3 B, C). To confirm this, we plotted the original linear and log-transformed response value as a function of the linear and log-transformed correct value (Fig. 3A) and tested the slope against a null value of zero using multi-level modeling, with participants as a random effect (table 3). There was significant linear dependence of the mean chosen value on the correct value for all conditions, for both the linear and log-transformed data. For all conditions, the rate of change of the conditional mean of the response value with respect to the correct value was greater than zero, for both the linear (symbolic multiplication: B = 0.9763, 95% C.I. [0.9656, 0.9871]; non-symbolic multiplication: B = 1.2427, 95% C.I. [1.1975, 1.2880]; symbolic division: B = 0.9868, 95% C.I. [0.9724, 1.0013]; non-symbolic division: B = 0.9306, 95% C.I. [0.8937, 0.9675]) and log-transformed data (symbolic multiplication: B = 0.9890, 95% C.I. [0.9810, 0.9970]; non-symbolic multiplication: B = 1.0731, 95% C.I. [1.0421, 1.0423]; symbolic division: B = 0.9809, 95% C.I. [0.9677–0.9941]; non-symbolic division: B = 0.9542, 95% C.I. [0.9238, 0.9845]). The log-transformed response and correct values appeared to better prepare the data for ANOVA since constant variation is assumed. Therefore, the logarithm of the response and correct value was used in all OM analyses.

**Table 3:** Linear increase of response value with correct value.

	*Linear Scale*			*Log scale*		
	*t*	Slope	95% CI	*t*	Slope	95% CI
*Multiplication*						
Symbolic	178.3*	.9763	.9656–.9871	242.7*	.9890	.9810–.9970
Non-symbolic	53.9*	1.2427	1.1975–1.2880	67.7*	1.0731	1.0421–1.0423
*Division*						
Symbolic	133.8*	.9868	.9724–1.0013	145.4*	.9809	.9677–.9941
Non-symbolic	49.4*	.9306	.8937–.9675	61.6*	.9542	.9238–.9845

Bonferroni corrected for multiple comparisons.

*p<.013.

### Contribution of both operands to response value

To determine whether participants considered both operands when choosing a response value, we performed multi-level multiple regression, separately for symbolic multiplication, non-symbolic multiplication, symbolic division, and non-symbolic division, using log-transformed values and participants as a random effect. For all conditions, there was a significant (*p*<.001) contribution of both operands to the mean response value and a significant rate of change of the conditional mean of the response value with respect to the first (op1) and second (op2) operands (symbolic multiplication: op1 *t* = 208.252, B = 0.9951, 95% C.I. [0.9857, 1.0045]; op2 *t* = 127.287, B = 0.9731, 95% C.I. [0.9581, 0.9881]; non-symbolic multiplication: op1 *t* = 51.852, B = 1.0990, 95% C.I. [1.0574, 1.1406]; op2 *t* = 29.389, B = 0.9962, 95% C.I. [0.9297, 1.0627]; symbolic division: op1 *t* = 131.116, B = 1.0105, 95% C.I. [0.9953, 1.0256]; op2 *t* = −81.827, B = −0.9733, 95% C.I. [−0.9966, −0.9500]; non-symbolic division: op1 *t* = 48.655, B = 0.9873, 95% C.I. [0.9475, 1.0271]; op2 *t* = −29.691, B = −0.9266, 95% C.I. [−0.9878, −0.8653]). The positive slopes for op2 in multiplication problems and negative slopes in division problems are consistent with the operations since a larger 2^nd^ operand in division would result in a smaller result value. Based on the conservative test of non-overlapping confidence intervals [36], [37], magnitudes of the slopes (absolute value of Β) where not significantly different between op1 and op2. These findings suggest that participants based their response on a combination of both operands and provide evidence against pure guessing.

Taken together, these results imply that participants did not consistently use a random guessing strategy. Rather, they relied on both operands, although perhaps not to an equal degree, to formulate a response. This supports the use of approximate calculation versus consideration of one operand. For symbolic problems, choices clearly peaked at the correct response. For non-symbolic problems, the pattern of results was more complex. However, the interaction between rank, range, and operation in non-symbolic problems implies that participants’ choices depended on the range of presented response alternatives in a given trial. Since the two ranges were presented in random order and participants were unaware of the low/high range design, the results are unlikely to be due to a completely non-numeric strategy. The increase of the mean chosen value as a function of the correct value, in all conditions, further supports this interpretation.

### Operational momentum effect

To investigate operational momentum, we looked at the response bias, defined as the difference between the log chosen and the log correct values. To test the influence of operation and notation on response bias, a 2-way repeated measures ANOVA was used. The interaction of operation and notation had a significant effect on response bias (*F*(1,15) = 16.023, *p* = .001, partial η^2^ = .516), qualifying significant main effects (operation: *F*(1,15) = 14.077, *p* = .002, partial η^2^ = .484; notation: *F*(1,15) = .297, *p* = .594, partial η^2^ = .019). Therefore, simple effects analysis was performed to see whether operation had an effect on mean response bias, separately for non-symbolic and symbolic notations.

#### Non-symbolic notation

**Figure 4:**
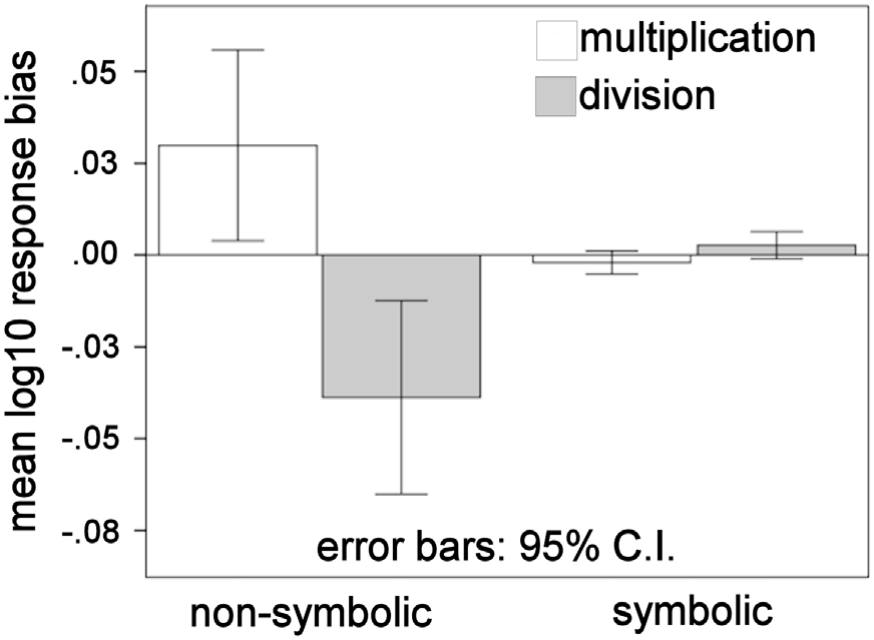
Operational momentum (OM) in non-symbolic, but not symbolic, notation. A significant response bias occurred for non-symbolic problems, indicating an OM effect. Symbolic response bias was not significant. Non-symbolic multiplication (light grey) problems were overestimated and division (dark gray) problems were underestimated. Response bias was calculated as the log_10_ response value – log_10_ correct value. Positive response bias indicates overestimation and negative indicates underestimation. Error bars represent 95% confidence interval (C.I.).

For non-symbolic problems, operation had a significant effect on response bias at the Bonferroni corrected *p*<.025 level (*F*(1,15) = 15.315, *p* = .001, partial η^2^ = .505). There was a significant difference in the mean log response bias between non-symbolic multiplication and division (M = 0.069, Bonferroni 95% C.I. [0.031, 0.106, p = .001]). To see if participants overestimated multiplication (mean log response bias>0) and underestimated division (mean log response bias<0), we performed one-sample t-tests against a null value of zero. We found that participants significantly overestimated multiplication problems (t(15) = 2.449, M = 0.02987, 95% C.I. [0.0039, 0.0559], *p* = .027) and underestimated division problems (t(15) = −3.136, M = −0.03879, 95% C.I. [−0.0652, −0.0124], *p* = .007). These results indicate that non-symbolic response bias is significantly influenced by operation. Consistent with our hypothesis, non-symbolic multiplication problems were overestimated while division problems were underestimated (Fig. 4).

#### Symbolic notation

For symbolic problems, operation did not have a significant effect on response bias (*F*(1,15) = 4.049, *p* = .063, partial η^2^ = .213). There was not a significant difference in the mean log response bias between symbolic multiplication and division (M = −.005, Bonferroni 95% C.I. [−0.01, 0.00], p = .063). The mean log response bias was not significantly different from zero in multiplication (t(15) = −1.365, M = −0.002, 95% C.I. [−0.005, 0.001], *p* = .192) or division (t(15) = 1.538, M = 0.003, 95% C.I. [−0.001, 0.006], *p* = .145) (Fig. 4).

## Discussion

Our primary goal in the present study was to determine if there was an OM effect for multiplication and division, like for addition and subtraction. Our second goal was to see if participants could use the ANS to perform non-symbolic multiplication and division, on larger quantities than previously studied. We hypothesized that participants could perform non-symbolic multiplication and division and would overestimate for multiplication, as they do for addition, and underestimate for division, as they do for subtraction. We found that participants could perform non-symbolic multiplication and division and their response patterns were consistent with use of the ANS. Participants significantly overestimated non-symbolic multiplication problems and underestimated non-symbolic division problems. Unlike symbolic addition and subtraction, for symbolic problems we observed no significant modulation of responses by operation. These findings expand the mathematical operations subject to response bias to include non-symbolic whole-number multiplication and division.

### Non-symbolic multiplication and division ability

To our knowledge, this is the first study to look at non-symbolic multiplication and division in adults. Previous research has focused on children and used smaller numbers (*e.g.* halving/doubling) [28], [29]. Based on the observed response pattern, participants likely used an approximate calculation based strategy for non-symbolic multiplication and division. If participants had ignored all numeric information and responded randomly, we would have seen a flat distribution across response choices (20% for each answer choice) both when the 2^nd^ and 4^th^ choices were correct (Fig. 2). This was obviously not the case, as seen in figure 2 and the significant interaction between rank and range in both notations. Alternatively, if participants had used a heuristic of choosing a relatively large number of dots for multiplication and small for division, we would have seen a distribution peaked at the high or low end of response choices, regardless of the response range presented. Again, this hypothesis was not supported by the data. Instead, participants overestimated multiplication when the 4^th^ of five answer choices was correct, but seemed to guess when the 2^nd^ lowest choice was correct. The reverse was found for division. Participants underestimated when the 2^nd^ choice was correct, but seemed to guess when the 4^th^ choice was correct. Since participants were not aware of the experimental design, let alone when they were answering in the low or high range, the consistent differences between ranges were most likely driven an approximate evaluation of the operands. Additionally, the mean chosen value increased with correct value (Fig. 3) and both the first and second operands independently contributed to the response value. Taken together, these findings indicate that participants were using calculation strategies that were influenced by operation. The influence of operation is consistent with the presence of OM in non-symbolic problems. Similar to addition and subtraction, the ANS might be used to solve whole-number non-symbolic multiplication and division. However, it should be noted that OM may be driven by non-calculation based strategies as was seen in infants [15]. Thus, the likely approximate calculation we have demonstrated is not a precondition for OM.

### Operational momentum effect in non-symbolic multiplication and division

This is the first study to look at OM in whole-number multiplication and division. Consistent with past research [13]–[15], we found an OM effect in non-symbolic calculations. Specifically, participants overestimated for multiplication and underestimated for division. Finding an OM effect is reminiscent of the whole-number bias [19], [20], as well as an extension of the ‘multiplication makes bigger, division makes smaller’ (than the original quantity) (MMBDMS) belief [22]. All of the response alternatives fit this belief, yet there was a bias towards over or underestimating. That is, over and above the predictions of the MMBDMS belief, we observed a modulation of mean chosen value by operation.

Three hypotheses for OM have been proposed: First, the compression hypothesis states that flawed decompression from the log scale results in response bias [13], [26]; however our data do not directly speak to this issue. Second, the attentional shifts hypothesis states that OM occurs as a result of left/right shifts of attention along a mental number line and a preference for outcomes in the whole-number biased [19], [20], [22] direction of the calculation [14]. Our findings support a preference for outcomes in the whole-number biased direction of the operation, although we did not test the role of attention. Finally, the heuristic (MMBDMS, in our study) hypothesis explains OM as using a rule of accepting more than the original operand for addition and less for subtraction [15], [19], [20], [22]. In principle, this could apply to multiplication and division. However, in our study the response choices were always numerically larger than both operands for multiplication and numerically smaller than the first operand for division. Therefore, any response would fit this rule. We found a bias within the presented choices even though they were equally likely to be chosen based on this heuristic. However, the influence of the range of presented response choices suggests that a similar heuristic could partially explain our findings. A combination of heuristic bias, similar to MMBDMS, and approximate calculation might best explain OM in whole-number multiplication and division. Based on the current results, we cannot rule out the possibility that approximate calculation was influenced by attentional shifts. Further research is needed to clarify the role of attention during approximate mental arithmetic.

The difference between low (4^th^ choice correct) and high (2^nd^ choice correct) range response choices suggests a more complex strategy than hypothesized for addition and subtraction. If participants had overestimated in multiplication (*i.e.* 2^nd^ correct: chosen 3^rd^, 4^th^, 5^th^; 4^th^ correct: chosen 5^th^) and underestimated in subtraction (*i.e.* 2^nd^ correct: chosen 1^st^; 4^th^ correct: chosen 1^st^, 2^nd^, 3^rd^), then a directionally biased approximate calculation hypothesis would explain our findings. Since participants were naïve to the study aims and manipulated factors (*e.g.* range), it remains to be seen what determines strategy choice in a given trial. If participants had chosen the largest response choice for multiplication (*i.e.* chosen 5^th^ in both 2^nd^ and 4^th^ correct) and smallest for division (*i.e.* chosen 1^st^ in both 2^nd^ and 4^th^ correct), then a modified MMBDMS heuristic hypothesis would explain our findings. Since they overestimated in multiplication only when the 4^th^ choice was correct and underestimated in division only when the 2^nd^ choice was correct, a combination of the two hypotheses might best explain our findings. Another possibility is that the ratio between the largest (multiplication) or smallest (division) response alternative and the correct outcome was too small for participants to exclude extreme results. For the high response range in multiplication and low in division, even extreme response alternatives were not considered too large (multiplication, high range) or small (division, low range), leading to a lack of tapering. However, this lack of tapering could be due to the operational momentum effect. That is, the operational momentum effect might be the reason why extreme values (too large for multiplication or small for division) did not seem extreme enough to exclude. More empirical data is needed to disentangle these possibilities. Thus, although our data cannot be explained by the traditional MMBDMS bias, a related heuristic strategy incorporating approximate calculation seems likely. This might be described as ‘multiplication makes relatively large, division makes relatively small’ (MRLDRS).

Although past OM research assumes that participants use a single strategy, they might use multiple strategies, especially for more difficult tasks. Symbolic arithmetic is thought to use global processes to evaluate solutions alongside fact retrieval [38], [39]. These biased global processes may originate from, or be exacerbated by, early educational methods [22]. Similarly, both heuristic evaluation (multiplication = relatively large answer; division = relatively small answer) (MRLDRS) and approximate calculation may be used for non-symbolic calculation and their interaction might explain OM. When a plausible response choice (dot array) based on the mentally represented approximate calculation is small relative to alternatives for multiplication (high range, 2^nd^ correct) or large for division (low range, 4^th^ correct), the approximate calculation and the MRLDRS heuristic evaluation lead to different response choices. When this conflicting information is present, accuracy is likely to decrease [40]. This is also in-line with the role of inhibitory control in numerical cognition [41], including OM [16].

In contrast to some research [14], [17], [18], we did not find OM in symbolic problems. The inclusion of the correct answer might have made performance too accurate to detect response bias. Studies finding OM in symbolic arithmetic have used approximate response methods, such as pointing to a line marked only with endpoint numbers [17], manual dot array generation [18], or jittering the correct result [14]. Though children may use the ANS to support symbolic arithmetic [1], [42], [43], reliance on rote verbal memory may limit ANS influence in adults [44]. Alternatively, regrouping performed in multiplication and division problems may prevent, and even reverse, OM. This explanation has been proposed for reverse OM in symbolic addition and subtraction [18]. A final possibility is that adult exposure to multiplication and division with rational numbers attenuates bias in whole-number symbolic calculation [22]. However, the demonstration of whole-number bias in adults suggests that directional bias is not fully corrected [19], [20]. Future research could use an approximate response method and a regrouping variable to understand whole-number symbolic OM.

### Limitations

This study has some limitations. Only five answer choices were presented, which might have put a ceiling or floor effect on response bias. Despite this, we were able to demonstrate OM. We also chose to keep the correct value identical across operations and to roughly match the 2^nd^ operands. Therefore, the size of the first operands was not matched between multiplication and division problems. However, if participants relied on the first operand we would expect, if anything, a reverse OM effect since division had larger first operands than multiplication.

### Conclusions

We have demonstrated that OM occurs in whole-number multiplication and division. This is the first time OM has been found in scalar operations. Additionally, we have shown that adults do not randomly guess or use a purely heuristic strategy, but rather use approximation, based on the operands, to perform non-symbolic multiplication and division. Non-symbolic multiplication problems are overestimated and non-symbolic division problems are underestimated. Interestingly, response patterns depend on the magnitude of the correct choice relative to the alternatives. These findings suggest that a combination of approximate calculation and an operationally dependent bias towards large or small quantities might explain OM. When multiple choices are given, response may depend on an interaction between approximate calculation and a heuristic evaluation. This interaction could reconcile these two previously proposed explanations. Future research should consider the use of multiple strategies, depending on difficulty and task design. However, regardless of the response strategy, the demonstration of OM in multiplication and division advances understanding of this phenomenon and shows that OM can be found in all whole-number arithmetic operations.
